# The association between gonadectomy and timing of gonadectomy, and the risk of canine cranial cruciate ligament disease: A systematic review and meta‐analysis

**DOI:** 10.1111/vsu.14197

**Published:** 2024-12-16

**Authors:** Daniel Low, Laura Costa, James Hawkesby, Ludovica Nardulli, Adelina Proteasa, Vasileios Vallios

**Affiliations:** ^1^ Frank. Pet Surgeons., IVC Evidensia Leeds UK; ^2^ Swift Referrals, IVC Evidensia Wetherby UK; ^3^ Small Animal Teaching Hospital The University of Liverpool Liverpool UK; ^4^ Chestergates Veterinary Specialists CVS Group Chester UK; ^5^ Faculty of Veterinary Medicine Ghent University Ghent Belgium

## Abstract

**Objective:**

To determine if gonadectomy in dogs is associated with the risk of cranial cruciate ligament disease (CrCLD) and to quantify the magnitude of the association.

**Study design:**

Systematic review and meta‐analysis.

**Sample population:**

Comparative studies with gonadectomized and entire dogs, with CrCLD as an outcome measure.

**Methods:**

A systematic search of the primary and gray literature was performed. The effect size of the outcome measure was defined as the OR and 95% CI. Subgroup analysis was performed with sex, breed, and age at gonadectomy. A pooled OR (95% CI) was generated from meta‐analysis of relevant studies. Certainty in the body of evidence was rated with the Grading of Recommendations Assessment, Development and Evaluation (GRADE) framework.

**Results:**

The literature search yielded 1398 results and 24 relevant studies were included for synthesis. Gonadectomized female (pooled OR = 2.293, 95% CI = 1.768–2.945) and male (pooled OR = 2.117, 95% CI = 1.665–2.691) dogs were both at increased odds of developing CrCLD in comparison with entire female and male dogs, respectively. Subgroup analysis showed that gonadectomy at 1 year or less was consistently associated with an increase in odds of developing CrCLD in both sexes. Overall certainty in the evidence was rated as moderate. All included studies were observational and no controlled trials were available.

**Conclusion:**

In data with moderate certainty, gonadectomy is associated with increased odds of developing CrCLD in both sexes, particularly in dogs gonadectomized at 1 year of age or less.

**Clinical significance:**

This study provides an estimate of the true effect size of gonadectomy on the odds of developing CrCLD, which may be useful for clinical decision making surrounding gonadectomy and the timing of gonadectomy.

**Abbreviations**
CrCLD: cranial cruciate ligament disease
GRADE: Grading of Recommendations Assessment, Development and Evaluation
LH: luteinizing hormone
NOS: Newcastle‐Ottawa scale
LOO: leave‐one‐out
OIS: optimal information size

## 1 INTRODUCTION

Cranial cruciate ligament disease (CrCLD) is a leading cause of pelvic limb lameness in the dog, with a reported prevalence of 0.56% to 12%.^1^, ^2^, ^3^ This disease is considered nontraumatic in nature,^4^, ^5^ with age, breed, body weight, sex, tibial plateau angle, proximal tibial morphology, and gonadectomy status having been previously reported to be associated with CrCLD risk.^1^, ^3^, ^6^, ^7^, ^8^, ^9^, ^10^, ^11^, ^12^, ^13^, ^14^ Gonadectomy has been associated with an increased risk of CrCLD in some studies^1^, ^3^, ^6^, ^7^, ^9^, ^10^, ^15^, ^16^, ^17^, ^18^, ^19^, ^20^ but other studies reported no association between gonadectomy and risk of unilateral or bilateral CrCLD.^8^, ^13^, ^21^ The impact of timing of gonadectomy is also unclear, with some studies reporting an increased risk of CrCLD with early gonadectomy^16^, ^22^, ^23^, ^24^, ^25^, ^26^ whereas other studies do not.^20^

A direct causal association between gonadectomy and CrCLD has yet to be demonstrated. Several mechanisms have been suggested. These include an increased risk of obesity, a greater tibial plateau angle,^27^ luteinizing hormone (LH) mediated pathways,^28^, ^29^ a longer lifespan and thus a lifetime risk of CrCLD,^30^ and a higher chance of being insured and therefore diagnosed with CrCLD.^6^, ^31^ Gonadectomy practices vary globally, with routine gonadectomy being more culturally acceptable and routinely performed in English‐speaking countries, whereas routine gonadectomy is illegal in Germany and Norway.^32^, ^33^ The majority of veterinarians in the United States recommend routine gonadectomy and believe that the benefits of this procedure outweigh its harms,^34^ with the majority of dogs in the United States having been castrated.^35^ Similarly, the United Kingdom public are generally supportive of routine gonadectomy,^36^ with thousands of gonadectomy procedures being performed in the United Kingdom annually.^6^, ^37^ Currently, there is no consensus on when gonadectomy should be performed in dogs; however, studies have demonstrated increased risks associated with gonadectomy at an early age, including an increased risk of nonorthopedic conditions.^26^ Unfortunately, there is no consensus definition of early gonadectomy in the literature, with varying definitions amongst studies, which therefore makes clinical decision making on the timing of gonadectomy more difficult for veterinarians.

Gonadectomy, and the timing of gonadectomy, thus represent potential modifiable risk factors in the development of CrCLD. Recommendations to clients vary greatly amongst veterinarians in the United Kingdom,^38^ possibly reflecting the heterogeneity of studies in the literature. Narrative reviews on this topic dominate in the literature^39^, ^40^, ^41^ but no systematic meta‐analyses are available, preventing high‐quality evidence‐based decisions from being made in clinical practice.

The objective of this study was to conduct a systematic review and meta‐analysis of the impact of gonadectomy on the risk of developing CrCLD amongst all dogs and their various subgroups. The null hypotheses were that dogs undergoing gonadectomy do not have a different risk of CrCLD from entire dogs, and that dogs undergoing early gonadectomy do not have a different risk of CrCLD from dogs undergoing late gonadectomy.

## 2 MATERIALS AND METHODS

This meta‐analysis was conducted and reported in accordance with the Preferred Reporting Items for Systematic Reviews and Meta‐Analyses (PRISMA 2020) statement Supporting Information (File S1), and was preregistered on the Open Science Framework on May 26, 2024, including the study rationale, hypotheses, inclusion criteria, data extraction processes, and planned analyses.^42^

### 2.1 Study inclusion and exclusion criteria

Gonadectomy was defined as orchiectomy in males and ovariectomy or ovariohysterectomy in females. Criteria for inclusion were (1) being a clinical study with dogs, (2) having the diagnosis of CrCLD as an outcome measure, although not necessarily a primary outcome measure, and (3) being a comparative study comparing entire and gonadectomized groups of dogs. Exclusion criteria were (1) secondary analyses of primary studies, (2) reporting of CrCLD as a composite outcome, (3) not being a comparative study, (4) studies of gonad‐sparing surgical procedures or nonsurgical methods of gonadectomy, and (5) review articles. Studies with overlapping data were excluded if reporting was insufficient to allow identification and removal of overlapping data, to prevent double counting, and to maintain data independence. Eligible studies were further excluded if relevant data were not reported or retrievable from the study, or if the authors were uncontactable for such data. Both prospectively and retrospectively conducted studies were eligible. There were no date or language restrictions. For synthesis, pooled gonadectomy data were analyzed separately for males and females. Breed‐specific subgroup analysis was performed if there were more than three studies evaluating a breed. Age at gonadectomy subgroup analysis was performed if there were more than three studies evaluating age at gonadectomy and risk of CrCLD.

### 2.2 Literature search

A literature search was performed on PubMed, CAB Abstracts, and Scopus on May 30, 2024. A gray literature search was performed on Proquest Dissertation and Theses Global and Web of Science on the same day. The literature search was repeated on July 7, 2024. Finally, an ancestral search of reference lists of eligible studies and review articles was conducted to identify relevant studies not found through prior literature searching.

The search strategy was developed through modification of a previously peer‐reviewed knowledge summary of a similar clinical question.^42^, ^43^ The search was conducted with variations of the keywords “dog” and “gonadectomy,” and the keyword “cruciate.” The full line‐by‐line search strategy is found in Supporting Information (File S2). The literature search process was then depicted graphically in a PRISMA flow diagram.^44^

### 2.3 Data extraction

Manual screening of relevant articles by title and abstract was performed by all authors working independently, with a shortlist of articles achieved by consensus. Full texts were retrieved by the lead author. All authors performed semiquantitative risk of bias assessment independently. Data extraction was performed by the lead author.

The outcome measure of interest in this study was the diagnosis of CrCLD. The method of CrCLD diagnosis was also recorded for risk of bias analysis. If odds ratios (ORs) or arm‐level data were not available from the study, the corresponding authors were contacted with a data request. If multiple studies were published using overlapping data from the same database or population, raw data were reviewed, if available, and the overlapping populations excluded. If not, the largest or most recent study was retained. In studies with overlapping data, the smaller study was excluded for meta‐analysis but retained for relevant subgroup analyses.

Exposure and outcome data were collected binarily. Other data were extracted for descriptive statistics and for meta‐regression, and included first author surnames, year of publication, country or region of study, study design, total eligible sample size, breeds studied (in breed‐specific studies), early gonadectomy definition, follow‐up duration, and receipt of external funding.

### 2.4 Risk of bias assessment

Study risk of bias was assessed with the semiquantitative Newcastle‐Ottawa scale (NOS) for nonrandomized studies,^45^ modified for this research question Supporting Information (File S3). Points were awarded for study design features that minimized risk of bias, with a maximum possible score of nine points. Interoperator agreement was assessed with Fleiss's kappa statistic. The final score was reported as a mean of the six independent assessments.

### 2.5 Statistical analysis

Odds ratios and 95% confidence intervals (CI) were retrieved or calculated with formulae in Supporting Information (File S4) and they indicated the effect sizes. Data were converted to the ln(OR) and corresponding standard error (SE_ln(OR)_) for statistical analysis. Covariate‐adjusted ORs were preferred over crude ORs. Odds ratios were defined as Odds_Gonadectomy_/Odds_Entire_ unless specified otherwise. For groups with zero events, a Haldane–Anscombe correction was applied.^46^

Heterogeneity between studies was assessed and quantified with the Cochran's Q test and the I^2^ statistic respectively. A *p* value of less than .05 was considered evidence of significant heterogeneity and I^2^ values below 25%, between 25% and 75%, and above 75% considered evidence of low, moderate, and high levels of heterogeneity respectively. In the event of a Cochran's Q test of *p* < .05 or an I^2^ more than 25%, a random effects model using a restricted maximum likelihood estimator was used instead of a fixed effects model. Meta‐regression was performed to explore the influence of covariates on the effect measure. An all subsets regression was performed to select the best model, with the Bayesian information criterion as the guiding metric. Funnel plots and Egger's test were used to assess risk of publication bias of studies. Sensitivity analyses were performed with the leave‐one‐out (LOO) method to assess the robustness of the pooled effect size to the removal of individual studies. The fail‐safe N was used to assess robustness of results arising from possible undetected publication or reporting biases. Finally, the certainty in the body of evidence was rated with the Grading of Recommendations Assessment, Development, and Evaluation (GRADE) framework, and achieved by consensus from all authors. Precision was assessed through evaluation of confidence interval magnitudes and the presence of overlap with the null effect, and through calculation of the optimal information size (OIS). All statistical analyses and data visualization were performed with *meta*,^47^ *metafor*,^48^ and *ggplot2* ^49^ in R version 4.4 and *pandas*,^50^ *numpy*,^51^ *matplotlib*,^52^ and *seaborn* ^53^ in Python version 3.10.

## 3 RESULTS

### 3.1 Study selection

**FIGURE 1:**
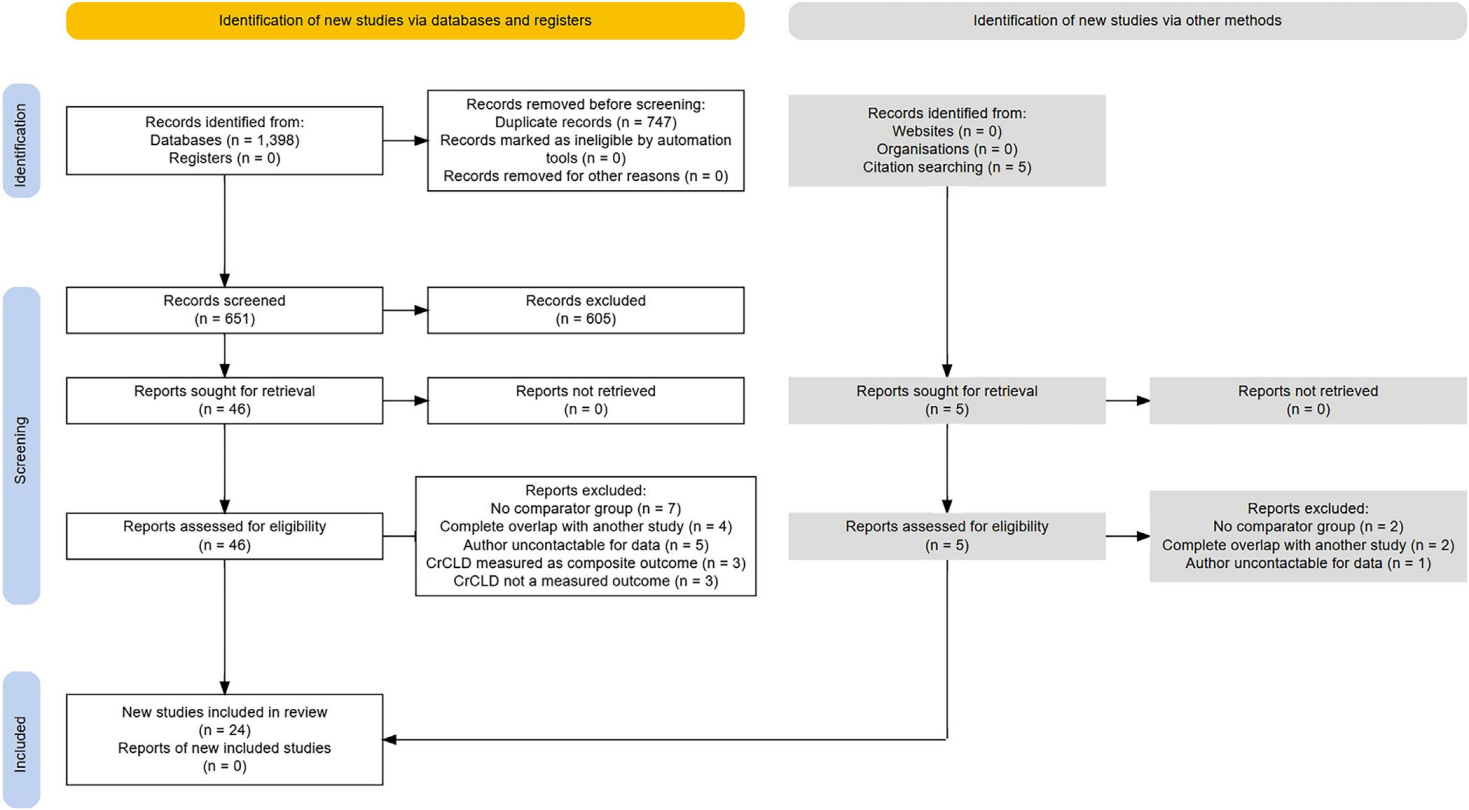
Preferred Reporting Items for Systematic Reviews and Meta‐Analyses (PRISMA) flow diagram outlining the literature search, screening, and inclusion process (https://www.eshackathon.org/software/PRISMA2020.html).

The initial literature search yielded 1398 articles from 1929 to 2024. Repeated literature searches yielded no new articles. After removal of duplicates, 651 articles were screened manually. From these, 46 articles met the inclusion criteria and were retrieved for full‐text screening. Five additional articles were identified and retrieved through citation searching. Of the 51 total articles undergoing full‐text screening for eligibility, one article was in Portuguese and the remaining 50 were in English. All 51 articles were reviewed by native speakers and 27 were excluded, leaving 24 studies for meta‐analysis (Figure 1).

Studies were excluded because of a lack of comparator group,^8^, ^11^, ^54^, ^55^, ^56^, ^57^, ^58^, ^59^, ^60^, ^61^ having complete overlap with another study,^20^, ^26^, ^62^, ^63^, ^64^, ^65^ because the authors did not respond to a data request,^17^, ^66^, ^67^, ^68^, ^69^ having CrCLD reported as a composite outcome,^24^, ^25^, ^70^ and not having CrCLD as a measured outcome.^71^, ^72^, ^73^ Toth and Siegel (2021)^19^ did not report arm‐level data in the publication but were able to provide these data on request and therefore the study was eligible for inclusion. Of the three studies reporting CrCLD as a composite outcome, two authors did not respond to a data request, and the remaining study was ineligible due to lack of a comparator group.^70^ Overall, 2/9 authors (22.2%) responded to a data request. Hart et al., 2014^22^ and Hart et al., 2016^23^ completely overlapped with Hart et al., 2020 (1)^74^ but both studies were retained as they each provided data on CrCLD risk stratified by age at gonadectomy and for specific breeds, and therefore were useful for subgroup analysis. Zink et al., 2023^75^ was retained after exclusion of the gonad‐sparing surgery subgroup.

### 3.2 Study characteristics

Study characteristics of the 24 included studies are presented in Table 1. Six studies were cohort studies^15^, ^22^, ^23^, ^74^, ^76^, ^77^ and the remaining 18 were case–control studies.^1^, ^3^, ^6^, ^7^, ^9^, ^10^, ^13^, ^16^, ^18^, ^19^, ^21^, ^75^, ^78^, ^79^, ^80^, ^81^, ^82^, ^83^ The studies were conducted in North America (*n* = 19), Europe (*n* = 5), or Asia (*n* = 1); one study was conducted across both North America and Europe. Eight studies reported breed‐specific data, of which five studies analyzed the Labrador retriever breed. Six studies studied CrCLD risk stratified by age at gonadectomy with Ekenstedt et al. (2017)^16^ defining early gonadectomy as gonadectomy performed at 1 year or younger, and the Hart series of studies^22^, ^23^, ^74^, ^76^, ^77^ stratifying age at gonadectomy into five nonuniform categories, <6 months, 6–11 months, 12–24 months, 2–8 years, and ≥9 years. Some studies applied inclusion criteria to their control groups, requiring controls to be at least 5 years of age,^78^ 7 years of age,^16^ or 8 years of age.^79^ Some studies applied exclusion criteria to their sample populations and excluded dogs less than 2 months of age,^3^ more than 2 years of age,^10^ more than 9 years of age,^22^, ^23^ or more than 11 years of age.^77^

**TABLE 1:** Summary statistics of included studies.

First author, year, reference	Country(s)	Study design	Total eligible sample size	Breed‐specific study	Labrador retriever	Golden retriever	German shepherd	Newfoundland	Rottweiler	Age at gonadectomy studied	Early gonadectomy definition	NOS score	Funding
Adams et al., (2011)^9^	United Kingdom	Case–control	1367	No						No		5	No
Baird et al., (2014)^78^	Europe and North America	Case–control	669	Yes	✔			✔	✔	No		4.67	Yes
Belanger et al., (2017)^15^	United States	Cohort	90 090	No						No		4.33	Yes
Cook et al., (2020)^79^	United States	Case–control	333	Yes	✔					No		4.67	No
Duval et al., (1999)^10^	United States	Case–control	973	No						No		3.5	No
Ekenstedt et al., (2017)^16^	United States and Canada	Case–control	311	Yes	✔					Yes	≤1 year, >1 year	5.67	No
Hart et al., (2014)^22^	United States	Cohort	2488	Yes	✔	✔				Yes	<6 m, 6–11 m, 12–24 m, 2–8 years	5	Yes
Hart et al., (2016)^23^	United States	Cohort	1118	Yes			✔			Yes	<6 m, 6–11 m, 12–24 m, 2–8 years	5.83	Yes
Hart et al., (2020) (1)^74^	United States	Cohort	11 528	Yes	✔	✔	✔		✔	Yes	<6 m, 6–11 m, 12–24 m, 2–8 years	5.5	Yes
Hart et al., (2020) (2)^76^	United States	Cohort	3141	No						Yes	<6 m, 6–11 m, 12–24 m, 2–8 years, ≥9 years	5	Yes
Hart et al., (2024)^77^	United States	Cohort	1492	Yes				✔		Yes	<6 m, 6–11 m, 12–24 m, 2–8 years, ≥9 years	5	Yes
Malek et al., (2020)^80^	United States and Canada	Case–control	112	No						No		3.5	Yes
Pegram et al., (2023)^6^	United Kingdom	Case–control	496 794	No						No		3.83	Yes
Powers et al., (2005)^21^	United States	Case–control	369	No						No		4.5	No
Sanchez‐Bustinduy et al., (2010)^81^	United Kingdom	Case–control	26	No						No		2.83	No
Sellon and Marcellin‐Little, (2022)^7^	United States	Case–control	1253	No						No		3.33	Yes
Seo et al., (2020)^82^	Korea	Case–control	274	No						No		5.33	Yes
Slauterbeck et al., (2004)^18^	United States	Case–control	3218	No						No		4.33	No
Taylor‐Brown et al., (2015)^1^	United Kingdom	Case–control	2828	No						No		5.5	Yes
Terhaar et al., (2020)^13^	United States	Case–control	412	No						No		5	Yes
Toth and Siegel, (2021)^19^	United States	Case–control	57	No						No		3.17	No
Wilke et al., (2006)^83^	United States	Case–control	163	Yes						No		4.33	Yes
Witsberger et al., (2008)^3^	North America	Case–control	1 243 681	No						No		3.67	No
Zink et al., (2023)^75^	North America	Case–control	5801	No						No		4.33	No

### 3.3 Risk of bias assessment

The mean NOS score, which was used to assess the methodological quality of the included studies, ranged from 2.83 to 5.83 Supporting Information (File S5) against a maximum score of 9. This indicated a moderate to high risk of bias across studies. Interobserver agreement was moderate (*κ* = 0.427).

### 3.4 Results of individual studies

Summary statistics of the studies that were included are presented in Supporting Information (File S6), showing arm‐level data, ORs, 95% CI, ln(OR), and SE_ln(OR)_ for each study, reported separately for females and males. Hart et al., (2014)^22^ and Hart et al., (2016)^23^ were excluded from analysis of aggregated data, due to complete data overlap with Hart et al., (2020) (2)^76^. Furthermore, Hart et al., (2020) (1),^74^ Hart et al., (2020) (2)^76^ and Hart et al., (2024)^77^ partially overlapped with Belanger et al., (2017).^15^ Each study had raw data available as supporting information, which was reviewed manually, allowing overlapping data to be excluded prior to analysis of aggregated data. A total of 1 854 685 dogs were available for aggregate analysis, which included 36 972 incident CrCLD cases and 773 128 gonadectomy procedures.

### 3.5 Results of meta‐analyses and subgroup analyses

**FIGURE 2:**
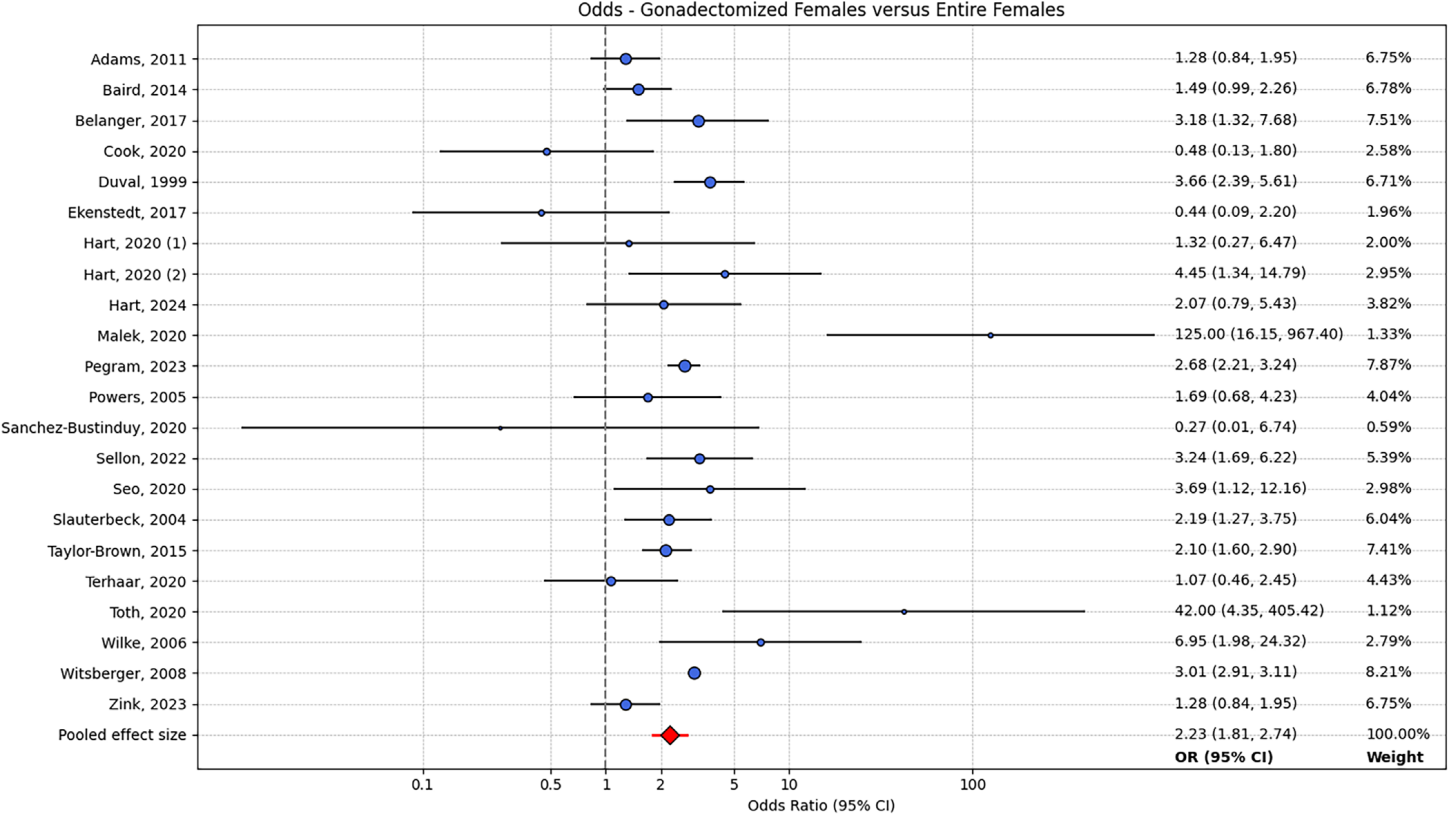
Forest plot showing OR (95% CI) of CrCLD for gonadectomized females versus entire females for each study, and the pooled OR (95% CI). OR, odds ratio; CI, confidence intervals. CrCLD, cranial cruciate ligament disease.

For the aggregated female group, Cochran's Q (Q = 94.53, *p* < .001) and the I^2^ statistic (86%) both showed evidence of heterogeneity. Twelve studies showed a significant increase in risk of CrCLD associated with gonadectomy, seven studies showed a nonsignificant increase in risk of CrCLD associated with gonadectomy, and three studies showed a nonsignificant decrease in risk of CrCLD associated with gonadectomy. Meta‐analysis with a random‐effects model returned a pooled effect size of 2.293 (95% CI: 1.768–2.945; Figure 2), indicating an increase in CrCLD risk associated with gonadectomy in female dogs.

**FIGURE 3:**
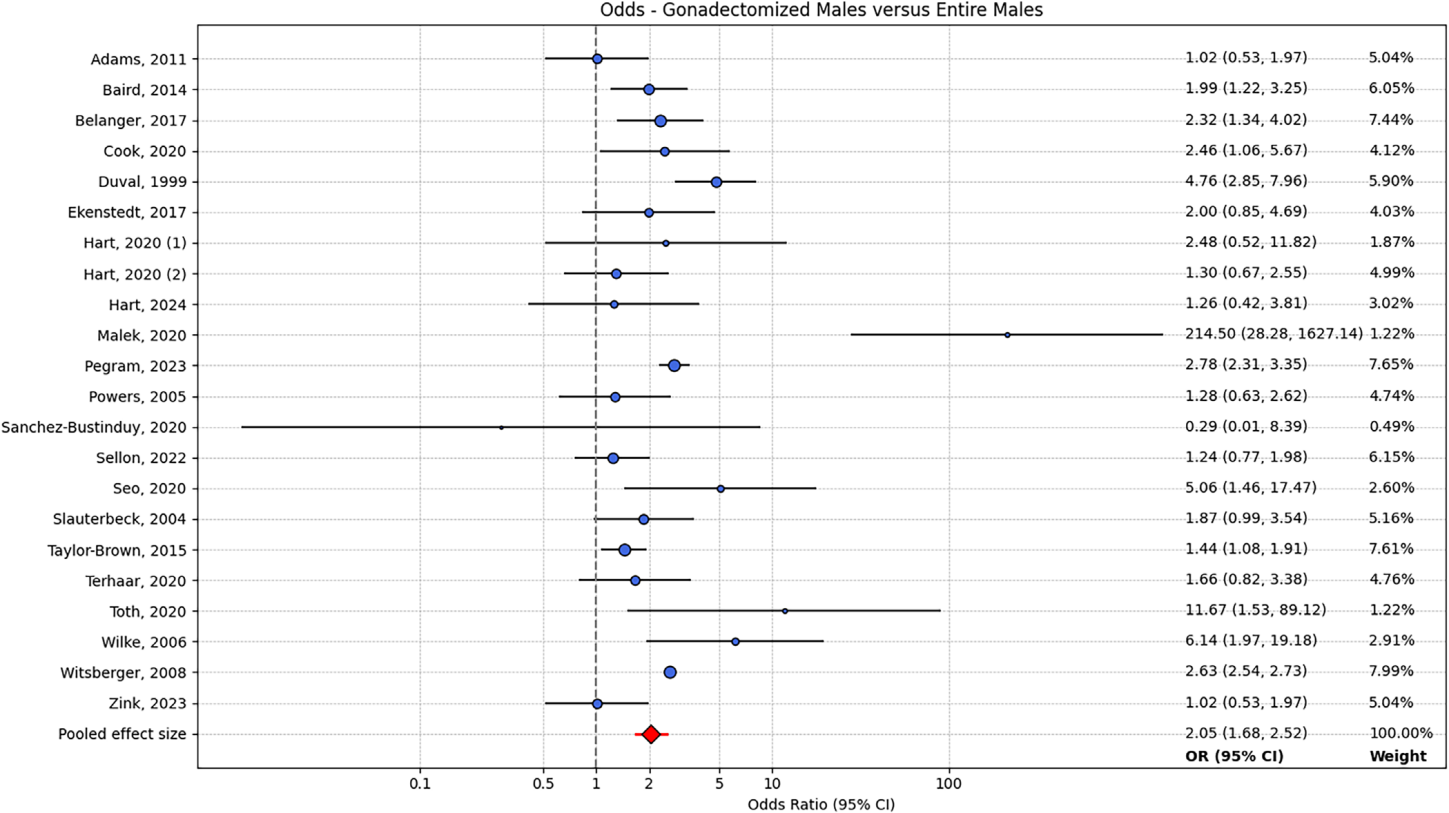
Forest plot showing OR (95% CI) of CrCLD for gonadectomized males versus entire males for each study, and the pooled OR (95% CI). OR, odds ratio; CI, confidence intervals; CrCLD, cranial cruciate ligament disease.

For the aggregated male group, Cochran's Q (Q = 104.6, *p* < .001) and the I^2^ statistic (87%), both showed evidence of significant heterogeneity. Eleven studies showed a significant increase in risk of CrCLD associated with gonadectomy, 10 studies showed a nonsignificant increase in risk of CrCLD associated with gonadectomy, and one study showed a nonsignificant decrease in risk of CrCLD associated with gonadectomy. Meta‐analysis with a random‐effects model returned a pooled effect size of 2.117 (95% CI: 1.665–2.691; Figure 3), indicating an increase in CrCLD risk associated with gonadectomy in male dogs.

**FIGURE 4:**
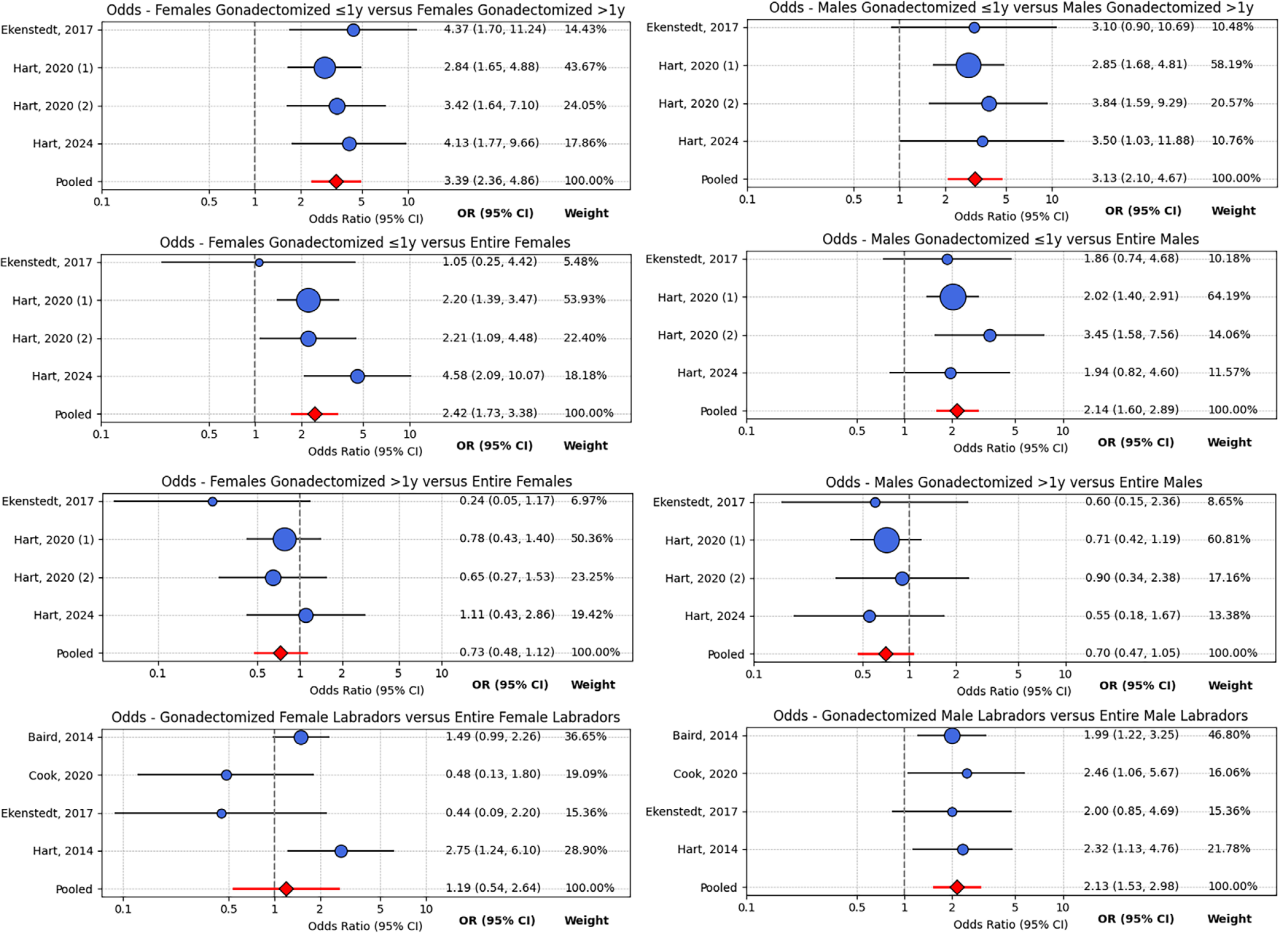
Forest plots showing OR (95% CI) of CrCLD of individual studies and a pooled OR (95% CI), by subgroup analysis. OR, odds ratio; CI, confidence intervals; CrCLD, cranial cruciate ligament disease.

Subgroup analysis was performed for females and males separately, by age at gonadectomy. Risk of CrCLD in dogs gonadectomized at 1 year of age or less was compared against dogs gonadectomized after 1 year (Comparison 1), and in dogs gonadectomized at 1 year of age against entire dogs (Comparison 2), and in dogs gonadectomized at more than 1 year of age against entire dogs (Comparison 3). Subgroup analysis by breed was only possible for the Labrador retriever, with four studies qualifying. Hart et al., (2014)^22^ and Hart et al., (2020) (1)^74^ both reported a dataset from a completely overlapping sample of Labrador retrievers, therefore Hart et al., (2014)^22^ was retained as the dataset for subgroup analysis. Risk of CrCLD in gonadectomized female Labrador retrievers was compared with entire female Labrador retrievers and the same pairwise comparison was performed for male Labrador retrievers (Table 2 and Figure 4).

**TABLE 2:** Subgroup analysis by age at gonadectomy and breed, showing synthesized odds ratios and 95% confidence intervals.

	Females	Males
Comparison 1 ($\frac{{\text{OR}}_{\text{gonadectomy}\leq 1y}}{{\text{OR}}_{\text{gonadectomy}>1y}}$)	**3.387 (2.363–4.855)**	**3.127 (2.096–4.665)**
Comparison 2 ($\frac{{\text{OR}}_{\text{gonadectomy}\leq 1y}}{{\text{OR}}_{\text{entire}}}$)	**2.415 (1.727–3.379)**	**2.138 (1.6–2.886)**
Comparison 3 ($\frac{{\text{OR}}_{\text{gonadectomy}>1y}}{{\text{OR}}_{\text{entire}}}$)	0.7335 (0.4819–1.116)	0.7047 (0.4727–1.051)
Labrador retriever	1.185 (0.5379–2.638)^a^	**2.133 (1.526–2.981)**

*Note*: Significant differences in odds ratios in bold.

^a^ Denotes meta‐analysis with a random effects model’ all other comparisons analyzed with a fixed effects model.

In the final meta‐regression model for female dogs, NOS score, funding, and study region were included as covariates. The NOS score was negatively associated with the effect measure, with an estimate of −0.8298 (95% CI: −1.552 to −0.107) and a *p* value of .03. In the final meta‐regression model for male dogs, sample size, funding, and a study across multiple regions were included as covariates. None of the covariates were associated with the effect measure.

Funnel plots for aggregated analysis of female and male dogs were examined and there was no visual evidence of publication bias Supporting Information (File S7). This was further supported by Egger's tests (*p* = .857 and *p* = .231 respectively).

Iterative removal of individual studies with the LOO method did not reveal any deviation in the pooled effect size for both the aggregated female and male groups Supporting Information (File S8). In other words, no individual studies were found to influence the pooled effect size disproportionately. The fail‐safe N values for the aggregated female and male groups were 4989 (*p* < .001) and 4112 (*p* < .001) respectively.

### 3.6 Certainty of evidence

The risk of bias, imprecision, inconsistency, indirectness, and publication bias were assessed using the GRADE framework. Risk of bias was judged to be moderate to high across studies and thus overall certainty was downrated accordingly. In conjunction with the moderately narrow confidence intervals and lack of overlap with the null effect in the aggregated synthesis for females and males, overall certainty was not downrated for imprecision. Consistency was judged to be low through the Cochran's Q and I^2^ statistic values obtained herein, and the varying point estimates of individual studies, and thus overall certainty was downrated for inconsistency. Direct applicability of all studies to the general population of dogs was low, as a referral population bias and regional bias were present through most studies, and thus certainty was downrated for indirectness. Publication bias was low to negligible, as assessed through funnel plot analysis; thus overall certainty was not downrated for publication bias. Based on these assessments, we rated the overall certainty in the evidence via the GRADE framework as moderate.

## 4 DISCUSSION

In this systematic review and meta‐analysis, we synthesized the existing literature quantitatively and demonstrated that gonadectomy is associated with increased odds of developing CrCLD in both female and male dogs. This association was also upheld when considering early gonadectomy, defined as that performed at 1 year of age or less; thus the null hypotheses were rejected. Notably, all qualifying studies were observational studies and provided level III and IV evidence only.^84^ All studies had moderate to high risk of bias, which should be considered when interpreting the pooled effect sizes.

This study synthesized aggregated data from over 1.8 million dogs and provided the most comprehensive estimate of CrCLD risk following gonadectomy. Point estimates of odds ratios reported in the literature vary greatly, which makes it challenging for veterinarians to counsel clients appropriately on the risks of gonadectomy, specifically in the context of CrCLD. This study provides evidence that gonadectomy increases the risk of CrCLD across all dogs, and that the practice of routine gonadectomy should be re‐evaluated, where CrCLD risk is concerned.^85^ In the context of policy making, breed‐specific legislation commonly requires gonadectomy to be performed as a condition of ownership.^86^, ^87^ In other legislatures, prepubertal gonadectomy may be encouraged^87^ or even made mandatory.^88^ These policies may have been implemented to promote public health but they can impose unintended consequences and costs on individual dogs and their owners.

Subgroup analysis by age at gonadectomy showed that odds of developing CrCLD are influenced by the age at which gonadectomy is performed, and that the pooled effect size only provides an estimate of average risk for the gonadectomized canine population as a whole. Notably, both female and male dogs gonadectomized at 1 year of age or less had similar odds of developing CrCLD compared to the general population of gonadectomized dogs when both groups were compared with entire dogs. This contrasts with previous studies that reported that early gonadectomy increased the risk of CrCLD.^16^, ^22^, ^23^, ^25^, ^26^ This discrepancy may be explained by our findings that gonadectomy at >1 year of age may result in reduced odds of developing CrCLD in both female and male dogs, although these differences were not statistically significant. These findings highlight that gonadectomy may have bidirectional effects on odds of developing CrCLD, depending on timing. In clinical practice, risk–benefit analysis of gonadectomy must therefore consider the patient's age and the intended age of gonadectomy. Future research should avoid dichotomous binning of dogs by gonadectomy status;^89^ instead, subgroup analysis by age of gonadectomy or considering cumulative exposure to gonadal hormones^90^ may be more methodologically sound.

It is well established that CrCLD is moderately heritable^4^ and the pooled effect size may have limited generalizability to certain breeds at very high or very low risk of CrCLD. Subgroup analysis in this study demonstrated that the association between gonadectomy and increased odds of CrCLD did not hold for gonadectomized female Labrador retrievers, who as a group, did not have increased odds of CrCLD compared to entire female Labrador retrievers. Breed differences have been previously explored^74^, ^77^ and it is likely that different breeds of dogs will be affected differently by removal of gonadal hormones, which may be an area for future study.

The association between gonadectomy and increased odds of developing CrCLD is likely complex and multifactorial. Gonadectomy has been shown to directly reduce resting energy requirement and thus increase the risk of obesity,^91^, ^92^ which is a known risk factor for development of CrCLD. Gonadectomized dogs have persistently elevated levels of LH, as a result of loss of feedback on the hypothalamic–pituitary‐gonadal axis, which has been suggested to have direct effects on nonreproductive LH‐receptor bearing tissues such as the cranial cruciate ligament.^28^, ^29^ Physeal development has been shown to be under the influence of gonadal hormones.^93^ A greater tibial plateau angle is a known risk factor for the development of CrCLD, and gonadectomized dogs have been shown to have a greater tibial plateau angle than entire controls.^27^, ^54^ Gonadectomized dogs may also represent a different socio‐economic subset of the canine population, and may have a longer lifespan or may be more likely to have pet insurance. Gonadectomized dogs may therefore have a greater lifetime risk of CrCLD or may be more likely to be diagnosed with CrCLD due to additional available funds or an increased likelihood of undergoing diagnostics.

The decision to perform gonadectomy in an individual animal should not be based solely on risk management of CrCLD as gonadectomy has been associated with increased risk of developing other orthopedic conditions, as well as nonorthopedic conditions including neoplasia, urinary tract disease, and behavioral issues.^74^ Conversely, gonadectomy may reduce the risk of mammary neoplasia in female dogs,^94^, ^95^ and effectively eliminates the risk of pyometra and testicular neoplasia in female and male dogs, respectively. Prepubertal gonadectomy also plays an important role in population control, in certain localities, and is associated with fewer surgical complications compared to gonadectomy performed in older dogs.^96^

This meta‐analysis was reported according to the PRISMA 2020 statement^97^ and preregistered to maximize transparency in reporting and to reduce the risk of bias.^98^ We included no language restrictions as this improves precision of meta‐analytical results over English‐language‐only meta‐analyses.^99^ We included comparative studies even when CrCLD and gonadectomy status were not being primarily investigated to minimize the risk of publication bias and selective reporting. There were eight studies that may have been included in the synthesis; however, data from these studies were unreported in the publication or unavailable from the authors. A 22.2% response rate to data requests was recorded in this meta‐analysis, below the 39.4% average across other scientific disciplines.^100^ Data availability is essential for ensuring reproducibility and for minimizing duplication of resources, and there is room for improvement in veterinary medicine. Risk of bias across studies was moderate to high, which is consistent with the fact that all studies were observational and most were retrospective in nature. Sources of heterogeneity were explored and likely to have arisen through differences in study populations across regions, as well as the analysis of different subgroups, as discussed earlier. Studies with a lower risk of bias were also associated with reporting a smaller effect size, and the varying study methodologies would have been another source of heterogeneity. Sensitivity analyses via the LOO method supported the robustness of the results herein, in that there were no studies that disproportionately skewed the pooled effect size. The NOS and GRADE framework were used to assess the risk of bias and certainty in the body of evidence respectively, and although these tools are recommended by the evidence‐based medicine community, they remain imperfect and subjective frameworks.^101^, ^102^ Nonetheless, the moderate interrater reliability that was obtained was within the range previously reported for cohort and case–control study assessment.^103^

As mentioned above, all studies included were observational, with methodological limitations, and provide levels III or IV evidence only. Some studies relied on owner or referring veterinarian reporting as a means of enrolling cases and controls. This may have led to misclassification bias whereby cases were incorrectly classified as controls, and vice versa. Across all studies, exposure data on gonadectomy status and age at gonadectomy was also collected secondarily based on owner reporting and, less frequently, referring veterinarian reporting. Again, these methods risk misclassification bias in that self‐reporting in epidemiological studies has not been shown to be a reliable means of collecting data.^104^ High heterogeneity between studies and varying inclusion and exclusion criteria limited the direct comparability of individual studies. Nonstandardized definitions of early gonadectomy also limited comparability between studies, and therefore, the strength of the meta‐analysis results from the timing of gonadectomy subgroup. The results of this meta‐analysis also reflect a regional and referral hospital bias, with the majority of studies conducted in North America and in referral hospitals, which may limit the applicability of results to the rest of the world and to the general canine population. Nonetheless, although the limitations of observational studies are recognized, it is unlikely that an adequately powered interventional study can be conducted for pragmatic reasons. Based on the OIS and the magnitude of the effect size determined herein, the sample size required to conduct a randomized controlled trial with α = 0.05 and β = 0.2 would be a minimum of 585 dogs in each intervention arm. Not only would it be logistically challenging to follow such a large number of dogs for years, but it is also ethically challenging to randomize client‐owned dogs to such an intervention.

In conclusion, this study systematically reviewed the literature on gonadectomy and CrCLD risk and showed that gonadectomy, in particular gonadectomy at 1 year of age or less, is associated with an increased risk of CrCLD in both female and male dogs. This study provides an estimate of the true effect size of gonadectomy on the odds of developing CrCLD, which may be useful for clinical decision‐making surrounding gonadectomy and timing of gonadectomy.

## FUNDING INFORMATION

Support for article processing charges was granted from IVC Evidensia.

## CONFLICT OF INTEREST

The authors declare no conflicts of interest related to this report.

## Supporting information


**Supplementary File S1.** PRISMA 2020 checklist.

**Supplementary File S2.** Line‐by‐line search strategy.

**Supplementary File S3.** Newcastle‐Ottawa scale for case–control and cohort studies.

**Supplementary File S4.** Formulae for odds ratio, 95% confidence interval, and standard error conversion.

**Supplementary File S5.** Mean NOS scores of individual studies.

**Supplementary File S6.** Summary statistics of included studies.

**Supplementary File S7.** Funnel plots for aggregated analysis of female (left) and male (right) dogs.

**Supplementary File S8.** Forest plots of sensitivity analyses of aggregated data from female and male dogs.
