# Supplementary material for: The association between gonadectomy and timing of gonadectomy, and the risk of canine cranial cruciate ligament disease: A systematic review and meta‐analysis
Source: Vet Surg. 2024 Dec 16;54(2):254–67. doi: 10.1111/vsu.14197 (PMC11830852; doi:10.1111/vsu.14197)
Supplement: Supplementary file 2 — Supplementary File S2. Line‐by‐line search strategy. [file VSU-54-254-s005.docx]

CAB Abstracts:

[[All: dog] OR [All: dogs] OR [All: hound*] OR [All: canin*] OR [All: canis] OR [All: bitch*] OR [All: pup] OR [All: pups] OR [All: pupp*]] AND [[All: spay*] OR [All: spey*] OR [All: ovariohysterectom*] OR [All: ovario-hysterectom*] OR [All: ovariectom*] OR [All: castrat*] OR [All: orchiectom*] OR [All: sterili*] OR [All: neuter*] OR [All: gonadectom*] OR [All: desex*] OR [All: de-sex*] OR [All: alter*] OR [All: fix] OR [All: fixi*] OR [All: fixe*]] AND [All: cruciate]

Pubmed:

("dogs"[MeSH Terms] OR "dogs"[All Fields] OR "dog"[All Fields] OR ("dogs"[MeSH Terms] OR "dogs"[All Fields]) OR "hound*"[All Fields] OR "canin*"[All Fields] OR "canis"[All Fields] OR "bitch*"[All Fields] OR "pup"[All Fields] OR "pups"[All Fields] OR "pupp*"[All Fields]) AND ("spay*"[All Fields] OR "spey*"[All Fields] OR "ovariohysterectom*"[All Fields] OR "ovario hysterectom*"[All Fields] OR "ovariectom*"[All Fields] OR "castrat*"[All Fields] OR “orchiectom*”[All Fields] OR "sterili*"[All Fields] OR "neuter*"[All Fields] OR "gonadectom*"[All Fields] OR "desex*"[All Fields] OR "de sex*"[All Fields] OR "alter*"[All Fields] OR "fix"[All Fields] OR "fixi*"[All Fields] OR "fixe*"[All Fields]) AND "cruciate*"[All Fields]

Scopus

TITLE-ABS-KEY ( ( dog OR dogs OR hound* OR canin* OR canis OR bitch* OR pup OR pups OR pupp* ) AND ( spay* OR spey* OR ovariohysterectom* OR ovario-hysterectom* OR ovariectom* OR castrat* OR orchiectom* OR sterili* OR neuter* OR gonadectom* OR desex* OR de-sex* OR alter* OR fix OR fixi* OR fixe* ) AND ( cruciate* ) )

Web of Science

TS=("dogs" OR "dog" OR "hound*" OR "canin*" OR "canis" OR "bitch*" OR "pup*" OR "pups" OR "pupp*") AND TS=("spay*" OR "spey*" OR "ovariohysterectom*" OR "ovario hysterectom*" OR "ovariectom*" OR "castrat*" OR “orchiectom*” OR "sterili*" OR "neuter*" OR "gonadectom*" OR "desex*" OR "de sex*" OR "alter*" OR "fix" OR "fixi*" OR "fixe*") AND TS="cruciate*"

ProQuest Dissertations & Theses Global

TI((dog OR dogs OR hound* OR canin* OR canis OR bitch* OR pup OR pups OR pupp*) AND (spay* OR spey* OR ovariohysterectom* OR ovario-hysterectom* OR ovariectom* OR castrat* OR orchiectom* OR sterili* OR neuter* OR gonadectom* OR desex* OR de-sex* OR alter* OR fix OR fixi* OR fixe*) AND cruciate)
