# Supplementary material for: The association between gonadectomy and timing of gonadectomy, and the risk of canine cranial cruciate ligament disease: A systematic review and meta‐analysis
Source: Vet Surg. 2024 Dec 16;54(2):254–67. doi: 10.1111/vsu.14197 (PMC11830852; doi:10.1111/vsu.14197)
Supplement: Supplementary file 3 — Supplementary File S3. Newcastle‐Ottawa scale for case–control and cohort studies. [file VSU-54-254-s003.pdf]

Data collection and Newcastle-Ottawa scale for case-control studies

Question	Explanatory notes	Score
Case selection		
1. Are cases adequately defined?<br>• Yes, with independent validation (1 point)<br>• Yes, with limited validation (0 points)<br>• No or not described (0 points)	• Are cruciate cases correctly defined?<br>○ Independent validation: diagnosis at referral hospital or primary centre conducting study<br>○ Limited validation: diagnosis based on referring vet, referring vet case history, or owner reporting<br>○ No: anything else	
2. Are cases representative?<br>• Yes (1 point)<br>• No or not described (0 points)	• Are cruciate cases representative of the general population of dogs with cruciate disease?<br>○ Yes: case sampling across referral and first-opinion population of dogs<br>○ No: referral population of dogs only, or not adequately described	
3. Are controls selected appropriately from the same population as cases?<br>• Community controls (1 point)<br>• Convenience sample (0 points)<br>• No or not described (0 points)	• Are controls representative of the general population of dogs?<br>○ Community controls: selected from across first-opinion +/- referral population<br>○ Convenience sample: selected from referral hospital population or from another convenience sample<br>○ No: anything else	
4. Are controls adequately defined?<br>• Yes, with independent	• Are controls (non-cruciate cases) correctly defined?<br>○ Independent validation: cruciate disease ruled out via physical exam and/or additional diagnostics	

<table>
<tr><td>validation (1 point)</td><td></td><td rowspan="3">Limited validation: cruciate disease ruled out via owner reporting or referring vet reporting
No: anything else</td><td rowspan="3"></td></tr>
<tr><td>• Yes, with limited validation (0 points)</td><td></td></tr>
<tr><td>• No or not described (0 points)</td><td></td></tr>
<tr><td colspan="4">Comparability</td></tr>
<tr><td colspan="2">5. Study accounts or controls for:</td><td rowspan="3">• Award 1 point if study accounts or controls for age at neutering (1 point) and any other clinical variables such as breed, bodyweight, age, insurance status etc (1 point)</td><td rowspan="3"></td></tr>
<tr><td>• Age at neutering (1 point)</td><td></td></tr>
<tr><td>• Any other clinical variables (1 point)</td><td></td></tr>
<tr><td colspan="4">Exposure</td></tr>
<tr><td colspan="2">6. How was exposure data collected?</td><td rowspan="4">• How did the study collect data on gonadectomy status (exposure variable)?
Primary record: gonadectomy performed at the centre conducting the study
Secondary record: records from another centre or from referring vet, or based on owner reporting
Not described</td><td rowspan="4"></td></tr>
<tr><td>• Primary record (1 point)</td><td></td></tr>
<tr><td>• Secondary record (0 points)</td><td></td></tr>
<tr><td>• Not described (0 points)</td><td></td></tr>
<tr><td colspan="2">7. Was exposure data collected in the same way for cases and controls?</td><td rowspan="4">• Did the study collect data on gonadectomy status in the same way for both cases (cruciate cases) and controls (non-cruciate cases)?
Yes
No</td><td rowspan="4"></td></tr>
<tr><td>• Yes (1 point)</td><td></td></tr>
<tr><td>• No (0 points)</td><td></td></tr>
<tr><td>• Not described (0 points)</td><td></td></tr>
<tr><td colspan="2">8. Was there incomplete exposure data in</td><td>• Was there incomplete gonadectomy data in either group? What</td><td></td></tr>
</table>

each group and was the proportion of incomplete data the same?		was the proportion of each group where gonadectomy data was incomplete and thus needing exclusion? Was the proportion the same?<br>○ No: complete gonadectomy data for all eligible individuals<br>○ Yes, but proportion incomplete similar: incomplete gonadectomy data present but proportion in case (cruciate) group and control (non-cruciate) group similar<br>○ Yes, and proportion incomplete different: incomplete gonadectomy data present and proportion in case (cruciate) group and control (non-cruciate) group different<br>○ Not described	
• No (1 point)			
• Yes, but proportion incomplete similar (1 point)			
• Yes, and proportion incomplete different (0 points)			
• Not described (0 points)			
		Total score (max 9)	

Data collection and Newcastle-Ottawa scale for cohort studies

Question	Explanatory notes	Score

Selection			
1. Is the exposed cohort representative?		• Is the exposed cohort (cohort of gonadectomised dogs) representative of the general population of dogs?<br>○ Yes, truly representative: cohort sampling across referral and first-opinion population of dogs<br>○ Yes, somewhat representative: cohort sampling across referral population of dogs only<br>○ No: narrow sample of dogs recruited for a specific reason or study objective, or not adequately described	
• Yes, truly representative (1 point)			
• Yes, somewhat representative (0 point)			
• No or not described (0 points)			
2. Is the non-exposed cohort comparable to the exposed cohort?		• Is the non-exposed cohort (cohort of sexually intact dogs) sampled in a similar way to the exposed cohort (cohort of gonadectomised dogs)?<br>○ Yes, same as exposed cohort: non-exposed cohort sampled identically to exposed cohort<br>○ No: non-exposed cohort sampled differently from exposed cohort, or not described	
• Yes, same as exposed cohort (1 point)			
• No or not described (0 points)			
3. How was exposure data collected?		• How did the study collect data on gonadectomy status (exposure variable)?<br>○ Primary record: gonadectomy performed at the centre conducting the study<br>○ Secondary record: records from another centre or from referring vet, or based on owner reporting<br>○ Not described	
• Primary record (1 point)			
• Secondary record (0 points)			
• Not described (0 points)			
4. Was the outcome of interest demonstrated to not be present at the start of the study?		• Did the study demonstrate that the outcome (cruciate disease) was not present at the start of the study?<br>○ Yes: longitudinal assessment was performed to rule out cruciate disease<br>○ No: anything else	
• Yes (1 point)			
• No or not described (0 points)			

Comparability			
5. Study accounts or controls for:		• Award 1 point if study accounts or controls for age at neutering (1 point) and any other clinical variables such as breed, bodyweight, age, insurance status etc (1 point)	
• Age at neutering (1 point)			
• Any other clinical variables (1 point)			
Outcome			
6. How was outcome data collected?		• How did the study collect outcome data (development of cruciate disease)?<br>○ Independent validation: diagnosis at referral hospital or primary centre conducting study<br>○ Limited validation: diagnosis based on referring vet, referring vet case history, or owner reporting<br>○ No: anything else	
• Independent validation (1 point)			
• Limited validation (0 points)			
• Not described (0 points)			
7. Was the follow-up period long enough?		• Did the study have a long enough follow-up period to sufficiently allow the outcome (cruciate disease) to manifest?<br>○ Yes: follow-up to patient death<br>○ No: anything else	
• Yes (1 point)			
• No (0 points)			
8. Was the follow-up methodology adequate?		• How successful was the study of following-up dogs? What was the rate of loss to follow-up?<br>○ Yes, complete follow-up data available: 100% rate of follow-up and methodology well-described<br>○ Yes, loss to follow-up less than 5%: 95-100% rate of follow-up, methodology well described, and losses explained<br>○ No, loss to follow-up more than 5%: <95% rate of follow-up, methodology well described, and losses explained	
• Yes, complete follow-up data available (1 point)			
• Yes, loss to follow-up less than 5% (1 point)			
• No, loss to follow-up more than 5% (0 points)			

● Not described (0 points)		○ Not described: methodology poorly described and/or losses not explained, or anything else	
		Total score (max 9)	
