# Supplementary material for: The association between gonadectomy and timing of gonadectomy, and the risk of canine cranial cruciate ligament disease: A systematic review and meta‐analysis
Source: Vet Surg. 2024 Dec 16;54(2):254–67. doi: 10.1111/vsu.14197 (PMC11830852; doi:10.1111/vsu.14197)
Supplement: Supplementary file 4 — Supplementary File S4. Formulae for odds ratio, 95% confidence interval, and standard error conversion. [file VSU-54-254-s002.pdf]

Formulae for odds ratio, 95% confidence interval, and standard error conversions

Let a, b, c, and d be defined as such:

	CrCLD (cases)	No CrCLD (controls)
Group A	a	b
Group B	c	d

$$OR_{A/B} = \frac{a \times d}{b \times c}$$

$$SE_{ln(OR)} = \sqrt{\frac{1}{a} + \frac{1}{b} + \frac{1}{c} + \frac{1}{d}} = \frac{SE_{OR}}{OR}$$

$$Lower\ CI = e^{ln(OR)} - 1.96(SE_{ln(OR)})$$

$$Upper\ CI = e^{ln(OR)} + 1.96(SE_{ln(OR)})$$

Haldane-Anscombe correction

$$OR_{A/B} = \frac{(a+0.5) \times (d+0.5)}{(b+0.5) \times (c+0.5)}$$

$$SE_{ln(OR)} = \sqrt{\frac{1}{a+0.5} + \frac{1}{b+0.5} + \frac{1}{c+0.5} + \frac{1}{d+0.5}}$$

ORs recalibration

$$OR_{recal} = \frac{OR_{original}}{OR_{new}}$$

$$SE_{ln(OR)} = \frac{ln(upper\ CI) - ln(lower\ CI)}{2\ x\ 1.96}$$

$$SE_{ln(OR)recal} = \sqrt{(SE_{ln(OR)original})^2 + (SE_{ln(OR)new})^2}$$
