# Supplementary figures and images for: The association between gonadectomy and timing of gonadectomy, and the risk of canine cranial cruciate ligament disease: A systematic review and meta‐analysis
Source: Vet Surg. 2024 Dec 16;54(2):254–67. doi: 10.1111/vsu.14197 (PMC11830852; doi:10.1111/vsu.14197)

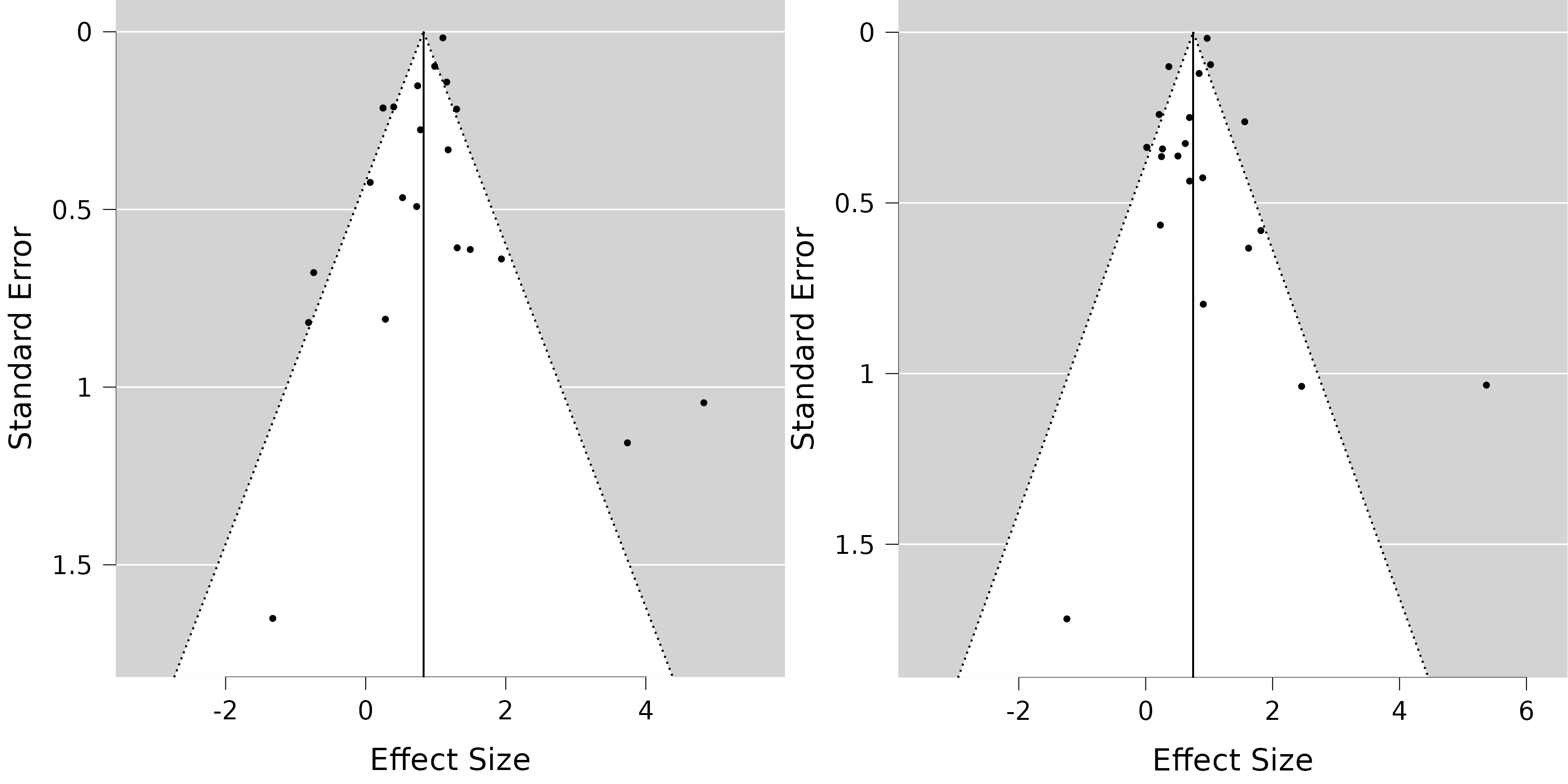

Supplement: Supplementary file 7 — Supplementary File S7. Funnel plots for aggregated analysis of female (left) and male (right) dogs. [file VSU-54-254-s006.png]

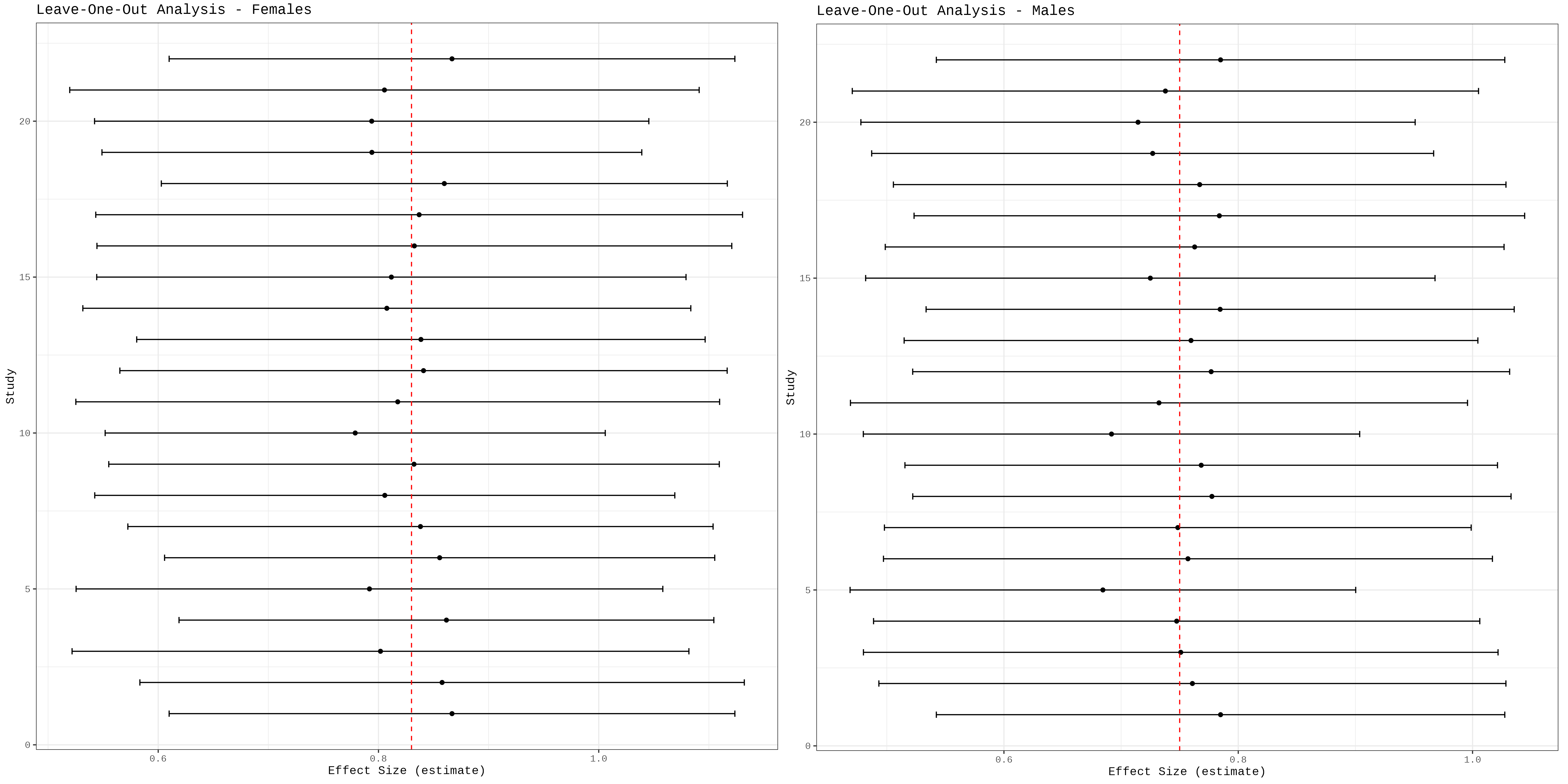

Supplement: Supplementary file 8 — Supplementary File S8. Forest plots of sensitivity analyses of aggregated data from female and male dogs. [file VSU-54-254-s001.png]
